# Acute High Fat Diet Consumption Activates the Mesolimbic Circuit and Requires Orexin Signaling in a Mouse Model

**DOI:** 10.1371/journal.pone.0087478

**Published:** 2014-01-23

**Authors:** Spring Valdivia, Anabela Patrone, Mirta Reynaldo, Mario Perello

**Affiliations:** Laboratory of Neurophysiology; Multidisciplinary Institute of Cell Biology (CONICET/CICPBA), La Plata, Argentina; Hosptial Infantil Universitario Niño Jesús, CIBEROBN, Spain

## Abstract

Overconsumption of palatable energy-dense foods has negative health implications and it is associated with obesity and several eating disorders. Currently, little is known about the neuronal circuitries activated by the acute ingestion of a rewarding stimulus. Here, we used a combination of immunohistochemistry, pharmacology and neuronal tracing analyses to examine the role of the mesolimbic system in general, and the orexin neurons in particular, in a simple experimental test in which naïve mice are allowed to spontaneously eat a pellet of a high fat diet (HFD) for 2 h. We found that acute HFD activates c-Fos expression in several reward-related brain areas, including the ventral tegmental area (VTA), nucleus accumbens, central amygdala and lateral hypothalamic area. We also found that: i- HFD-mediated orosensory stimulation was required for the mesolimbic pathway activation, ii- acute HFD differentially activates dopamine neurons of the paranigral, parabrachial pigmented and interfascicular sub-regions of the VTA, and iii- orexin neurons of the lateral hypothalamic area are responsive to acute HFD. Moreover, orexin signaling blockade, with the orexin 1 receptor antagonist SB-334867, reduces acute HFD consumption and c-Fos induction in the VTA but not in the other mesolimbic nuclei under study. Finally, we found that most orexin neurons responsive to acute HFD innervate the VTA. Our results show that acute HFD consumption recruits the mesolimbic system and that the full manifestation of this eating behavior requires the activation of orexin signaling.

## Introduction

Consumption of palatable energy-dense food is a rewarding experience for most animals, including human beings. An emerging literature suggests that the current environment, where inexpensive palatable foods are easily accessible, promotes overconsumption of calories that can lead to obesity or eating disorders [1]–[4]. In terms of the neurobiological bases of this phenomenon, some evidence suggests that the hedonic brain circuits that drive consumption based on the rewarding properties of foods can override brain circuits that drive food intake depending on energy store levels, leading to intake of calories beyond energetic and nutritional requirements [1]–[4]. However, the neuronal circuitries activated by the acute ingestion of a rewarding stimulus are currently unclear. The mesolimbic system participates in the reinforcing and motivational effects of several rewarding stimuli [5]. This system consists of dopamine neurons that project from the ventral tegmental area (VTA) to various forebrain areas including the accumbens (NAc) as well as the central amygdala (CeA), prefrontal cortex, hippocampus and hypothalamus [6], [7]. The VTA is a neuroanatomically and functionally complex brain area that contains diverse neuronal populations that may play distinct roles in reward-related behaviors [8], [9]. It is currently unclear whether the mesolimbic system plays a role in the hedonic-driven food consumption [7], [10], [11]. The mesolimbic system is activated in response to palatable foods, and dopamine release in the NAc augments the drive to obtain food rewards [12]–[14]. However, NAc dopamine depletion alone does not alter feeding, and pharmacological blockade of dopamine receptors in the NAc affects motor behavior and has no effects on food intake [13], [15]. The NAc shell has been implicated in hedonic eating since it sends projections to neurons of the lateral hypothalamic area (LHA) controlling food intake [12], [16]. In the LHA, orexin (also known as hypocretin)-producing neurons seem to be under a tonic inhibition that can be relieved by activation of reward pathways [16]–[18]. LHA orexin neurons regulate the VTA dopamine neurons and have been implicated in food reward modulation [17]–[23]. To our knowledge, it has not been explored whether the VTA-NAc-LHA pathway plays a role in acute consumption of a highly palatable food. Here, we used a combination of immunohistochemistry, pharmacology and neuronal tracing analyses to examine the role of the neuronal populations of the VTA-NAc-LHA pathway during acute HFD consumption.

## Methods

### Animals and diets

Adult (2–3 month old) C57BL6/J mice were generated at the animal care facility of the Multidisciplinary Institute of Cell Biology (IMBICE). Male mice were housed under a 12-h light/dark cycle (lights on at 07:00 a.m.) and with regular chow and water available *ad lib*. This study was carried out in strict accordance with the recommendations in the Guide for the Care and Use of Laboratory Animals of the National Research Council, USA [24], and all efforts were made to minimize suffering. The protocol was approved by the Institutional Animal Care and Use Committee of the IMBICE (approval ID 10-0113).

**Figure 1:**
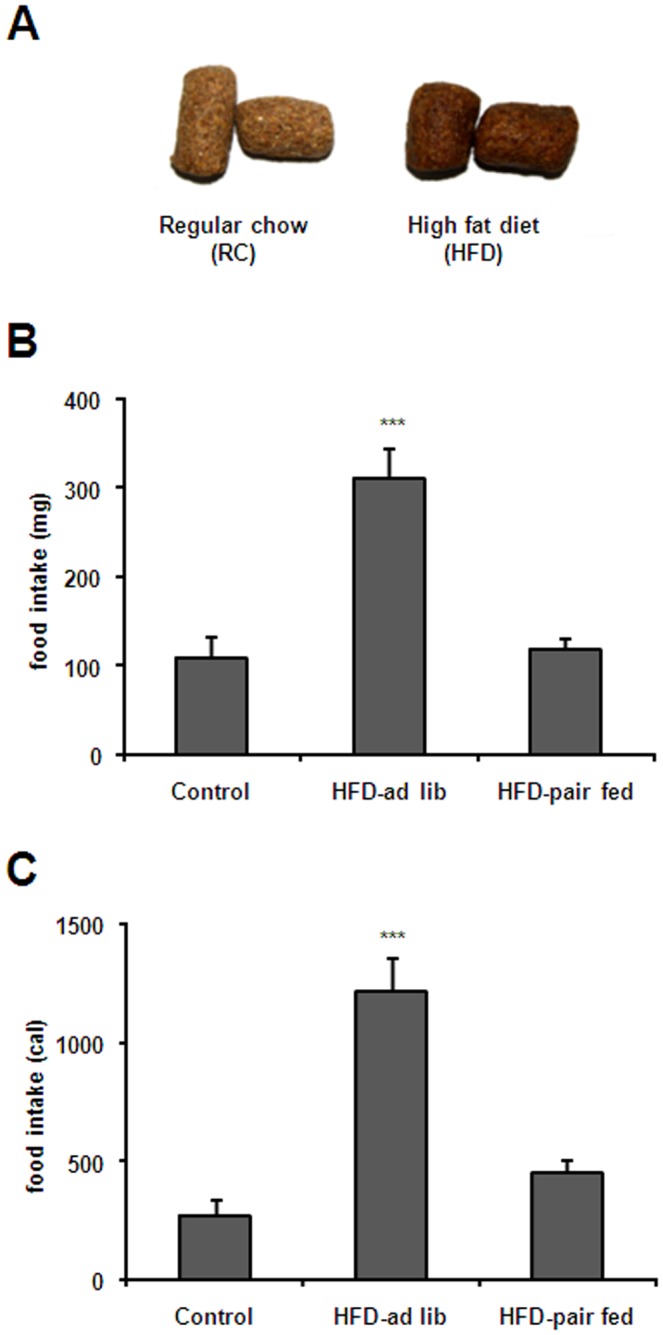
Exposure to HFD induces food intake in mice. Panel **A** shows images of regular chow and HFD pellets used in the current study. Panels **B** and **C** show 2 h food intake in control, HFD-*ad lib* and HFD-pair-fed groups expressed either in mg or calories, respectively. Values are the mean±SEM. ***, p<0.001 vs. control and HDF-pair-fed group.

Both regular chow (RC) and HFD were provided by Gepsa Feeds (Grupo Pilar, Pilar, Buenos Aires, Argentina, www.gepsa.com). RC pellets provided 2.5 kcal/g energy and its composition was as follows: carbohydrate 28.8%, proteins 25.5%, fat 3.6%, fibers 27.4%, minerals 8.1% and humidity 6.7% (http://www.gepsa.com/institucional/es/labAutoclave.asp). HFD pellets were custom-prepared and provided 3.9 kcal/g energy. The composition of the HFD was as follows: carbohydrate 22.5%, proteins 22.8%, fat 21.1%, fibers 23.0%, minerals 5.6% and humidity 5.0%. The main fat components of HFD pellets were monounsaturated fatty acids (44.7%), saturated fatty acids (29.8%) and polyunsaturated fatty acids (20.9%), among others. Importantly, color, texture and overall appearance of custom-prepared HFD pellets were similar to those of RC pellets (Figure 1A).

### Experimental protocols

In the morning of the experimental day (9.00 a.m.), *ad lib* fed mice were exposed to a pellet of either RC or HFD for 2 h (n = 16 and 14, respectively). In order to distinguish potential effects of the HFD itself and those of higher food intake, a pair-fed control group consisting of mice exposed to amounts of HFD similar to those eaten by mice exposed to RC was included (n = 10). The cumulative food intake was recorded 2 h after food exposure.

In order to perform acute intragastric feeding, either RC or HFD were resuspended in water and administered via plastic feeding tubes for oral rodent gavage (cat# FTP-20-38, Instech Solomon Laboratories). Oral gavage procedure was performed by trained personnel as we have previously done in the past [25]. The distance from the tip of the animal's head to the last rib was used as a reference for the length of the gavage tube in order to avoid stomach perforation. Mice were made accustomed to handling and trained on the procedure daily for at least 4 days prior to experimentation to reduce stress. Briefly, the mouse's head was extended backwards to create a straight line through the neck and esophagus. The gavage tube was placed in the diastema of the mouth and then gently advanced along the upper palate until the end of the esophagus was reached. Mice (n = 4 per group) were given one infusion intragastrically containing 310 mg of diet as a slurry in a total volume of 1 mL. Of note, control group received ∼0.77 kcal and HFD group received ∼1.21 kcal. After infusion, the tube was gently removed following the same angle as insertion. Then, mice were returned the home cage and anesthetized 2 h later.

In another experiment, orexin 1 receptor antagonist SB-334867 (Tocris, cat.# 1960) was used to block orexin signaling. This compound was i.p. injected at a dose of 5 µg/g body weight in 200 µL saline at 8.30 a.m. A similar dose of SB-334867 has been shown to affect high-fat intake in many models while it does not affect food intake of freely-available RC [25]–[28]. Thirty min after treatment, *ad lib* fed mice injected with either vehicle or SB-334867 were exposed to a pellet of HFD for 2 h (n = 10 and 8, respectively). Here, an extra pair-fed control group consisting of vehicle-injected mice exposed to amounts of HFD similar to those eaten by SB-334867-treated mice was included in order to distinguish the specific effects of SB-334867 (n = 9). The cumulative food intake was recorded 2 h after food exposure.

### Tracing Studies

In order to label LHA neurons innervating the VTA, the retrograde tracer FluoSpheres (Red Fluorescent, Invitrogen cat.# F8793) was stereotaxically microinjected into the VTA (n = 6). The placement coordinates were: AP -3.52, L 0.42, and V 4.27 mm to bregma. These coordinates were initially obtained from the Paxinos atlas [29] and then modified based on pilot studies. The injector was positioned with a micromanipulator and an 800 nL volume of 2.5% FluoSpheres in saline was injected via a 33-gauge injector. The injector was left in place for 10 min following the injection to allow the tracer to diffuse away from the injection site. After 6 days for retrograde transport of the FluoSpheres, *ad lib* fed mice were exposed to a pellet of HFD for 2 h and then anesthetized. Control group was excluded from this experiment because this study was specifically performed to test if orexin neurons responsive to acute HFD, as indicated by the presence of c-Fos signal, send their projections to the VTA. Of note, stereotaxic microinjections of FluoSpheres in the VTA resulted in 3 missed injections in which the tracer did not diffuse to all three VTA subdivisions. These mice were excluded from the analysis.

### C-Fos immunostaining

Two hour after spontaneous food intake or acute intragastric feeding, mice were anesthetized and perfused with formalin as previously described [30]. Then, brains were removed, immersed in 20% sucrose overnight and coronally cut at 20 µm into three equal series on a sliding cryostat. C-Fos immunostaining was performed as described before [30]. Briefly, sections were pretreated with H_2_O_2_, treated with blocking solution and incubated with anti-c-Fos antibody (Calbiochem/Oncogene, cat# PC38, 1∶10,000) for 2 days at 4°C. Then, sections were incubated with biotinylated donkey anti-rabbit antibody (Jackson ImmunoResearch Laboratories, cat# 711-065-152, 1∶1,000) for 1 h, and with Vectastain Elite ABC kit (Vector Laboratories, cat# PK-6200) for 1 h, according to manufacturer's protocols. Then, visible signal was developed with 3-3′-diaminobenzidine (DAB)/Nickel solution (Sigma Aldrich, cat# 32750), which generated a black/purple precipitate. Sections were sequentially mounted on glass slides and coverslipped with mounting media. Bright-field images were acquired with a Nikon Eclipse 50i and a DS-Ri1 Nikon digital camera. Adobe Photoshop CS2 image editing software was used to adjust levels, contrast and brightness.

### Double c-Fos and tyrosine hydroxylase (TH) immunostaining

Independent series of brain sections were used for double immunostaining for c-Fos and TH (n = 6 per group). After c-Fos immunostaining was completed, brain sections were incubated 48 h with an anti-TH antibody (Sigma-Aldrich, cat# T2928, 1∶4,000) and then sequentially incubated with biotinylated donkey anti-mouse antibody (Vector Laboratories, cat# BA-9200, 1∶2,000) and Vectastain Elite ABC kit as detailed above. Finally, visible signal was developed by incubation with DAB solution, showing a brown precipitate. Sections were sequentially mounted on glass slides and coverslipped with mounting media. Bright-field images were obtained as described above.

### Double c-Fos and orexin immunostaining

Orexin immunostaining was performed using an anti-orexin antibody (Phoenix Pharmaceuticals, cat# H-003-30, 1∶20,000). In one experiment, c-Fos immunostained brain sections were used to detect orexin-IR signal using brown chromogenic immunostaining as indicated above (n = 6 per group). In samples from tracing studies, c-Fos immunostained brain sections were used for detection of orexin-IR signal using fluorescent immunostaining. After washing, brain slices were incubated overnight with the anti-orexin antibody. Next day, sections were incubated with fluorescent donkey anti-rabbit Alexa 488 antibody (Invitrogen, cat# A21207, 1∶1,000) during 2 h, mounted, and coverslipped in a fluorescence mounting solution-containing DAPI. Results were visualized using either fluorescence (orexin and FluoSpheres) or bright-field light (c-Fos) sources and images were obtained as described above.

### Single orexin and double TH/orexin immunostaining within the VTA

Brain sections containing the VTA were used for detection of orexin-IR signal using the anti-orexin antibody and black/purple chromogenic immunostaining as indicated above (n = 6 per group). Independent series of brain sections were used first for detection of TH-IR signal using the anti-TH antibody and brown chromogenic immunostaining, and then for orexin immunostaining using the anti-orexin antibody and black/purple chromogenic immunostaining as indicated above (n = 6 per group).

### Quantitative neuroanatomical analysis

To determine the total number c-Fos-immunoreactive (IR) cells in each brain region, cells containing distinct nuclear black/purple precipitate were quantified in one out of three complete series of coronal brain sections. Anatomical limits of each brain region were identified using a mouse brain atlas [29]. Total c-Fos-IR cells in each brain region were estimated in sections between bregma 0.86 and 1.54 mm for the NAc; between bregma −0.70 and −1.70 mm for the CeA and between bregma −0.82 and −1.94 mm for the LHA. For the analysis, the VTA was subdivided into paranigral nucleus (PN), parabrachial pigmented area (PBP) and interfascicular nucleus (IF), according to previous descriptions, and analyzed between bregma −3.08 and −3.80 mm [8], [31], [32]. Data were expressed as c-Fos-IR cells per coronal section. Blind quantitative analysis was performed independently by two observers, in 10–14 animals per experimental condition. In the case of c-Fos/TH co-staining, TH-IR was confined to the perikarya and dendrites, thus allowing visualization of the nucleus with or without black/purple label for c-Fos. Total TH-IR neurons and TH-IR neurons with nuclei positive for c-Fos were counted in the different VTA sub-regions. Orexin-IR was also confined to the perikarya and dendrites, which allowed visualization of the nuclei with or without black/purple label for c-Fos. Total orexin-IR neurons and orexin-IR neurons with nuclei positive for c-Fos were counted in each side of the third ventricle. Then, double immunostaining analyses were expressed as the total number of neurons positive for c-Fos observed in each brain region. The estimation of total TH-IR cells in the VTA and total orexin-IR cells in the LHA were corrected for double counting, according to the method of Abercrombie [33], where the ratio of the actual number of neurons to the observed number is represented by T/T+h where T = section thickness, and h = the mean diameter of the neuron. For this, cell diameter was quantified, of at least 40 cells in each brain area and experimental group, using the software ImageJ.

For quantitative estimates of orexin-IR fibers density within each VTA sub-region, images of VTA sections labeled for orexin were acquired with a 100X objective lens, in comparable areas and under the same optical and light conditions. To estimate the orexin-IR fiber density, microphotographs were transformed to 8-bit greyscale images, and the mean optical density (OD) for each image was measured using ImageJ software. The OD measurement for each image is the mean grey value of the pixels, with a 256 grayscale value as a reference. Nonspecific background was determined in the dorsal midbrain, where no orexin-IR fibers were detected. The OD value for each VTA sub-region corresponds to the mean orexin-IR fibers density minus the respective background level of the sample. For quantitative estimation of the number of somata of TH-IR neurons contacted by orexin-IR fibers (axonal boutons), sections double labeled for TH and orexin were visualized using a 100X objective lens and images were acquired as described above. Then, total TH-IR neurons and TH-IR neurons contacted by orexin-IR fibers were counted per high magnification picture and results were expressed as total TH-IR neurons contacted by orexin-IR fibers in each VTA sub-region. In tracing studies, triple-, double- and single-labeled neurons were counted, and relationships were expressed as percentage of either double- or single-labeled cells.

### Statistical analyses

Data were expressed as mean±SEM. One-way ANOVA followed by the Newman Keuls test was used to compare food intake or quantitative analysis of neuroanatomical data. Correlation between 2 h food intake and c-Fos-IR cells in the IF sub-region of the VTA was performed by linear regression analysis using a general linear model. Significant differences were considered when p<0.05.

## Results

### Acute HFD activates c-Fos expression in reward-related brain areas

**Figure 2:**
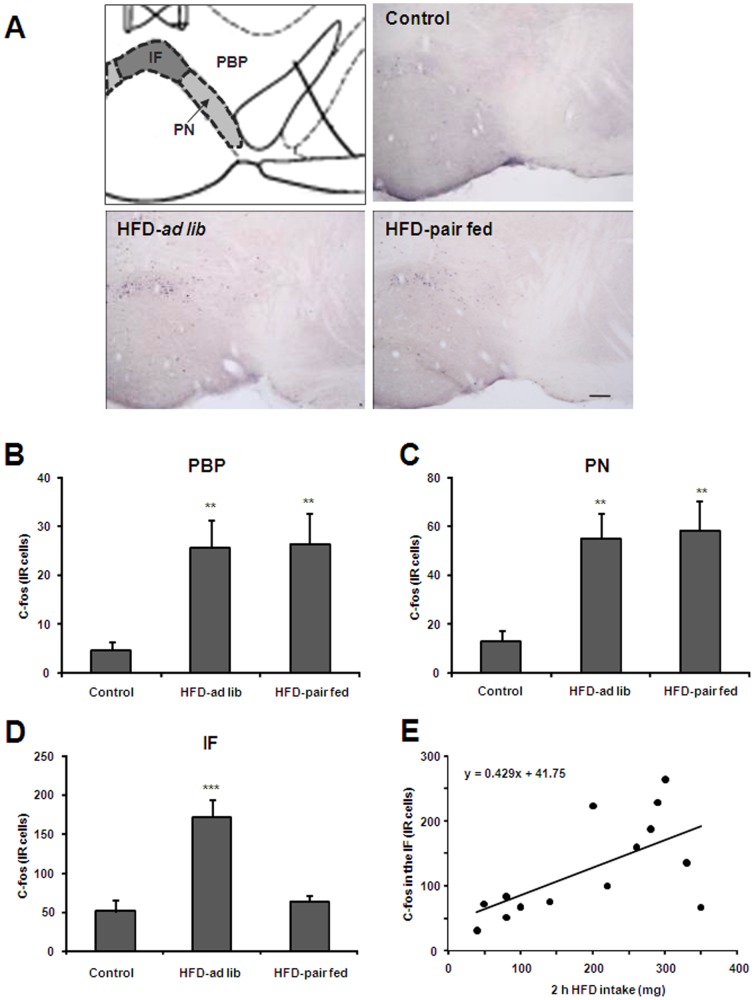
Acute HFD activates c-Fos in specific sub-regions of the VTA. Panel **A** shows a schematic diagram of VTA sub-regions in a coronal section of the mouse brain (upper left) and representative microphotographs of c-Fos (black/purple signal) immuno-staining in the VTA of control (upper right), HFD-*ad lib* (bottom left) and HFD-pair-fed (bottom right) groups. Scale bar: 100 µm. Panels **B-D** show quantitative analysis of c-Fos staining in specific PBP (B), PN (C) and IF (D) sub-regions of the VTA. Histograms depict the total number of c-Fos-IR neurons, expressed as cells per coronal section, for each experimental group. Values are the mean±SEM. **, p<0.01 vs. control group. ***, p<0.001 vs. control group. Panel E shows the correlation between 2 h food intake and c-Fos-IR cells in the IF sub-region of the VTA in HFD-*ad lib* group (r = 0.628). Each point represents a measurement from a single animal.

**Figure 3:**
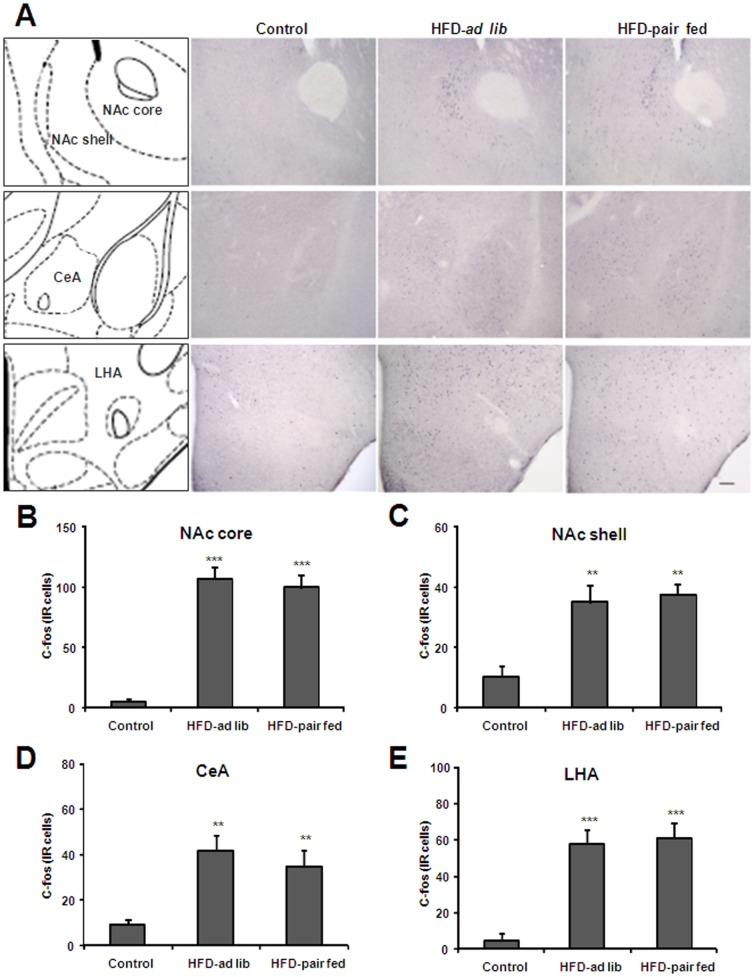
Acute HFD activates c-Fos in specific nucleus of mesolimbic pathway. Panel **A** shows a schematic diagram of the brain regions under study in a coronal section of the mouse brain (left column) and representative microphotographs of c-Fos immuno-staining of control, HFD-*ad lib* and HFD-pair-fed groups. Upper, middle and bottom line of images show the NAc (core and shell), the CeA and the LHA, respectively. Scale bar: 100 µm. Panels **B-E** show quantitative analysis of c-Fos staining in the NAc core (B), NAc shell (C), CeA (D) and LHA (E). Histograms depict the total number of c-Fos-IR neurons, expressed as cells per coronal section, for each experimental group. Values are the mean±SEM. **, p<0.01 vs. control group. ***, p<0.001 vs. control group.

In order to map brain areas responsive to acute HFD, *ad-lib* fed mice were exposed to a pellet of either RC or HFD for 2 h and then processed for immunostaining. Total food intake was significantly higher in animals exposed to HFD as compared to those exposed to RC (311±35 vs. 110±23 mg respectively, p<0.001, Figure 1B). Total food intake in HFD pair-fed mice was 119±15 mg. Since RC and HFD were not isocaloric, a significant difference of caloric intake between the experimental groups was also observed [F(2,34) = 22.64, P<0.0001]. Total caloric intake for control, HFD-*ad lib* and HFD-pair-fed groups was 275±67, 1216±146 and 455±56 cal, respectively (Figure 1C). Thus, caloric intake in HFD-*ad lib* mice remained significantly higher as compared to caloric intake observed in both control and HFD-pair-fed groups (P<0.001) while caloric intake did not differ between control and HFD-pair-fed groups (P>0.05). The density of observed c-Fos-IR cells is outlined in Table 1, which also includes the abbreviations used in the figures and throughout the text. C-Fos-IR signal within the hypothalamus was moderate and enriched in some nuclei such as the PVH, DMN and LHA. However, the higher number and strongest intensity of c-Fos-IR cells were observed in several nuclei of the mesolimbic pathway, including the VTA, NAc and CeA. Thus, a detailed quantitative analysis was performed in these areas. Acute HFD induced a significant change in the number of c-Fos-IR cells within the PN [F(2,30) = 6.826, P = 0.0036], PBP [F(2,29) = 8.099, P = 0.0016] and IF [F(2,26) = 19.03, P<0.0001] sub-regions of the VTA (Figure 2A). The number of c-Fos-IR cells in the PN and PBP sub-regions of both HFD-*ad lib* and HFD-pair-fed groups was significantly higher as compared to the number observed in control group (Figure 2B–C). In the PN sub-division, 12.8±4.8, 55.2±10.4 and 58.2±14.4 c-Fos-IR cells were detected in control, HFD-*ad lib* and HFD-pair-fed groups, respectively (P<0.01 vs. control group). In the PBP sub-division, 4.8±1.6, 25.6±5.6 and 26.4±6.4 c-Fos-IR cells were detected in control, HFD-*ad lib* and HFD-pair-fed groups, respectively (P<0.01 vs. control group). In contrast, the number of c-Fos-IR cells in the IF sub-region of HFD-*ad lib* mice was significantly higher as compared to levels found in both control and HFD-pair-fed groups. In particular, 51.2±14.4, 171.2±22.4 and 64.0±7.2 c-Fos-IR cells were detected in control, HFD-*ad lib* and HFD-pair-fed groups, respectively (P<0.001 vs. control group) (Figure 2D). Moreover, food intake positively correlated with the total number of c-Fos-IR cells in the IF sub-region of the VTA in HFD-*ad lib* mice (Figure 2E). A significant HFD-induced change in the number of c-Fos-IR cells was also found in other brain areas belonging to the mesolimbic pathway (Figure 3A) including the shell and core sub-divisions of the NAc [shell: F(2,26) = 13.59, P<0.0001; core: F(2,24) = 59.99, P<0.0001] as well as the CeA [F(2,28) = 9.012, P<0.001] and LHA [F(2,26) = 31.37, P<0.0001]. In particular, 5.7±2.4, 107.0±9.4 and 99.6±10.7 c-Fos-IR cells were detected in the NAc core of control, HFD-*ad lib* and HFD-pair-fed groups, respectively (P<0.001 vs. control group, Figure 3B) while 8.6±3.4, 35.0±5.7 and 37.7±3.4 c-Fos-IR cells were detected in the NAc shell of control, HFD-*ad lib* and HFD-pair-fed groups, respectively (P<0.01 vs. control group, Figure 3C). In the CeA, 9.3±2.2, 42.2±6.7 and 35.2±7.1 c-Fos-IR cells were detected in control, HFD-*ad lib* and HFD-pair-fed groups, respectively (P<0.01 vs. control group, Figure 3D). In the LHA, 4.8±4.1, 57.7±8.3 and 61.3±8.1 c-Fos-IR cells were detected in control, HFD-*ad lib* and HFD-pair-fed groups, respectively (P<0.001 vs. control group, Figure 3E). Thus, these data indicate that acute HFD activates neuronal populations of the mesolimbic pathway.

**Table 1:** Relative density of c-Fos-IR in the central nervous system of experimental groups^1^.

	Control	HFD-*ad lib*	HFD-pair-fed
Arcuate nucleus-ARC	–	+/–	+/–
Dorsomedial nucleus-DMN	+	+	+
Lateral hypothalamic area-LHA	–	++	++
Paraventricular nucleus-PVH	–	+	+
Ventral tegmental area-VTA	–	++	+
Nucleus Accumbens-Acc	–	++	++
Caudate Putamen	–	–	–
Central Amygdala-CeA	–	+	+
Dorsal raphe nucleus-DR	–	+	–
Parabrachial nucleus-PBN	–	+	+
Area postrema-AP	–	–	–
Dorsal motor nucleus of the vagus-DMNV	+	+	+
Nucleus of the solitary tract-NTS	+	+	+

1 Qualitative estimates of c-Fos-IR were made by considering both signal strength and the number of labeled cells: ++, high density; +, moderate density; +/–, inconsistent visualization.

### Acute gastric tube feeding with HFD fails to activate c-Fos expression in the mesolimbic pathway

In order to determine if the HFD-induced increase of c-Fos expression in the mesolimbic pathway required oral stimulation, *ad lib* fed mice were intragastrically administered with HFD or RC. As compared to the control group, intragastric administration of HFD failed to activate c-Fos expression in most nuclei of the mesolimbic pathway, including the IF sub-region of the VTA (40.1±10.1 *vs*. 51.2±14.4 IR-cells, respectively), NAc shell (10.6±3.4 *vs*. 7.2±1.4 IR-cells, respectively), CeA (9.3±2.2 *vs*. 11.1±2.6 IR-cells, respectively) and LHA (10.8±4.1 *vs*. 13.2±2.7 IR-cells respectively). Thus, it seems that oral stimulation is important in eliciting the rewarding effect of HFD.

### Acute HFD activates c-Fos expression in TH-IR cells of VTA

**Figure 4:**
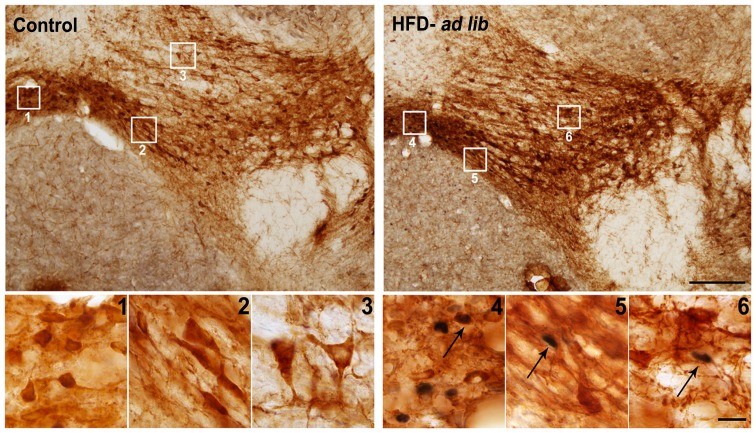
Acute HFD activates c-Fos in TH-IR neurons of specific sub-regions of the VTA. Panels show representative photomicrographs of brain sections subjected to double immunohistochemistry using anti-TH (brown staining) and anti-c-Fos (purple/black staining) antibodies. Left and right panels show low magnification images from a mouse of control and HFD-*ad lib* group, respectively. Panels 1 to 6 show in higher magnification the areas marked in low magnification images. Panels 1 and 4 show the IF, panels 2 and 5 show the PN, and panels 3 and 6 show the PBP sub-region of the VTA. Arrows point to dual-labeled cells. Scale bars, 200 µm (low magnification), 20 µm (high magnification).

To determine if dopamine neurons of the VTA were activated by acute HFD consumption, double immunohistochemistry for c-Fos/TH was performed (Figure 4). Cytoarchitectonic features of TH-IR cells within the different sub-regions of the VTA were similar to those described for the rat [8]. TH-IR cells in the PN were relatively homogeneous, mostly medium sized, medium to dark stained and semi-laterally oriented. TH-IR cells in the PBP were large, medium to low stained and with no uniform orientation. The IF is localized just dorsal to the interpeduncular nucleus; TH-IR cells within this nucleus were small, medium to dark stained and densely packed along the midline. The total number of TH-IR cells within VTA sub-regions was not affected by acute HFD: 746±91, 1433±153 and 365±42 TH-IR neurons were estimated in the PN, PBP and IF subdivisions of the VTA of control mice, respectively; 816±76, 1363±126 and 352±51 TH-IR neurons were estimated in the PN, PBP and IF subdivisions of the VTA of HFD-*ad lib* fed mice, respectively; and 856±95, 1416±161 and 402±57 TH-IR neurons were estimated in the PN, PBP and IF subdivisions of the VTA of HFD-pair-fed mice, respectively. Although the total number of TH-IR cells within the VTA sub-regions was unchanged, a significant change of the number of TH-IR cells positive for c-Fos was found in the PN [F(2,17) = 4.448, P = 0.0279], PBP [F(2,17) = 9.179, P = 0.0020] and IF [F(2,17) = 8.995, P = 0.0022] sub-divisions of the VTA. In the PN and PBP sub-regions, the number of TH-IR neurons positive for c-Fos of both HFD-*ad lib* and HFD-pair-fed groups was significantly higher as compared to the control group (P<0.01). Quantitative analysis showed that 13±1, 62±15 and 52±11 TH-IR neurons of the PN sub-division were positive for c-Fos in control, HFD-*ad lib* and HFD-pair-fed groups, respectively, while 4±4, 17±4 and 23±6 TH-IR neurons of the PBP sub-division were positive for c-Fos in control, HFD-*ad lib* and HFD-pair-fed groups, respectively. Interestingly, c-Fos-IR nuclei were exclusively found in TH-IR cells of the PN and PBP sub-regions of the VTA in all experimental groups. The number of TH-IR neurons positive for c-Fos in the IF sub-region of the VTA of HFD-*ad lib* mice was significantly higher as compared to numbers found in both control and HFD-pair-fed groups (P<0.01). Quantitative analysis of the IF sub-region indicated that 14±5 and 21±2 TH-IR neurons were positive for c-Fos in control and HFD-pair-fed groups, respectively, while 51±8 TH-IR neurons were positive for c-Fos in the HFD-*ad lib* group. HFD-induced increase of c-Fos affected both TH-IR and non-TH-IR cells of the IF sub-region of the VTA. In particular, TH-IR cells positive for c-Fos represented 44.0±4.2% of all c-Fos-IR cells in the IF of HFD-*ad lib* groups. Thus, acute HFD exclusively affects TH-IR cells in the PN and PBP, while it affects both TH-IR and non-TH-IR cells of the IF sub-region of the VTA.

### Acute HFD activates c-Fos expression in orexin-IR neurons of the LHA

**Figure 5:**
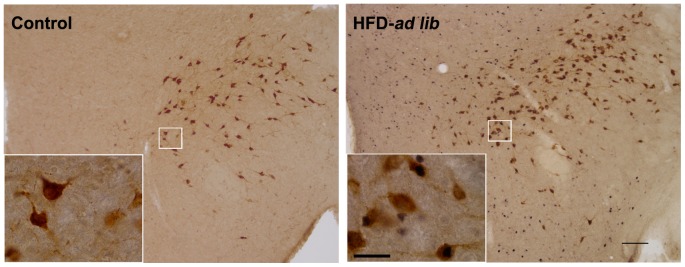
Acute HFD activates c-Fos in orexin-IR neurons of the LHA. Panels show representative photomicrographs of brain sections subjected to double immunohistochemistry using anti-orexin (brown staining) and anti-c-Fos (purple/black staining) antibodies. Left and right panels show images from mice of control and HFD-*ad lib* groups, respectively. Inserts show in higher magnification the areas marked in low magnification images. Arrows point to dual-labeled cells. Scale bars, 200 µm (low magnification), 20 µm (high magnification).

To determine if orexin neurons of the LHA were activated by acute HFD consumption, double immunohistochemistry for c-Fos/orexin was performed (Figure 5). Acute HFD failed to affect the total number of orexin-IR cells since 1510±162, 1428±149 and 1486±165 orexin-IR cells were detected in the LHA of control, HFD-*ad lib* and HFD-pair-fed groups, respectively (P>0.05). A significant difference of the number orexin-IR cells positive for c-Fos was found within LHA of the different experimental groups [F(2,9) = 13.01, P = 0.0022]. Quantitative analysis indicated that 16±15, 298±68 and 358±57 of orexin-IR neurons were positive for c-Fos in control, HFD-*ad lib* and HFD-pair-fed groups, respectively (P<0.01 vs. control group). The distribution of the orexin-IR neurons positive for c-Fos did not show any particular topography within the LHA. These data indicated that acute HFD activates orexin-IR neurons of the LHA, independently of the amount eaten.

### Orexin signaling blockade reduces acute HFD consumption and c-Fos induction in the VTA

**Figure 6:**
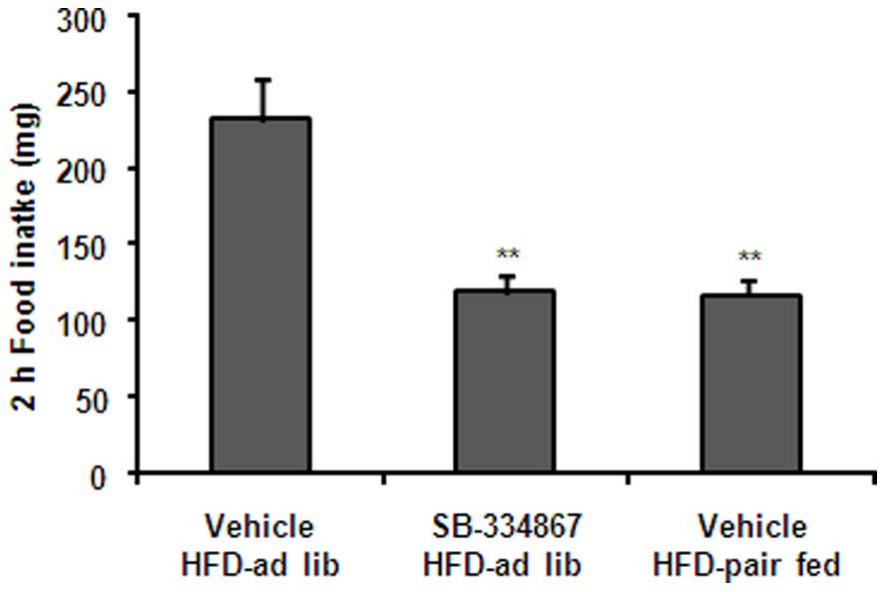
Orexin signaling blockade reduces spontaneous consumption of HFD. Figure shows 2-HFD-*ad lib*, SB334867-HFD-*ad lib* and vehicle-HFD-pair-fed groups. Values are the mean±SEM. **, p<0.01 vs. vehicle HDF-*ad lib* group.

**Figure 7:**
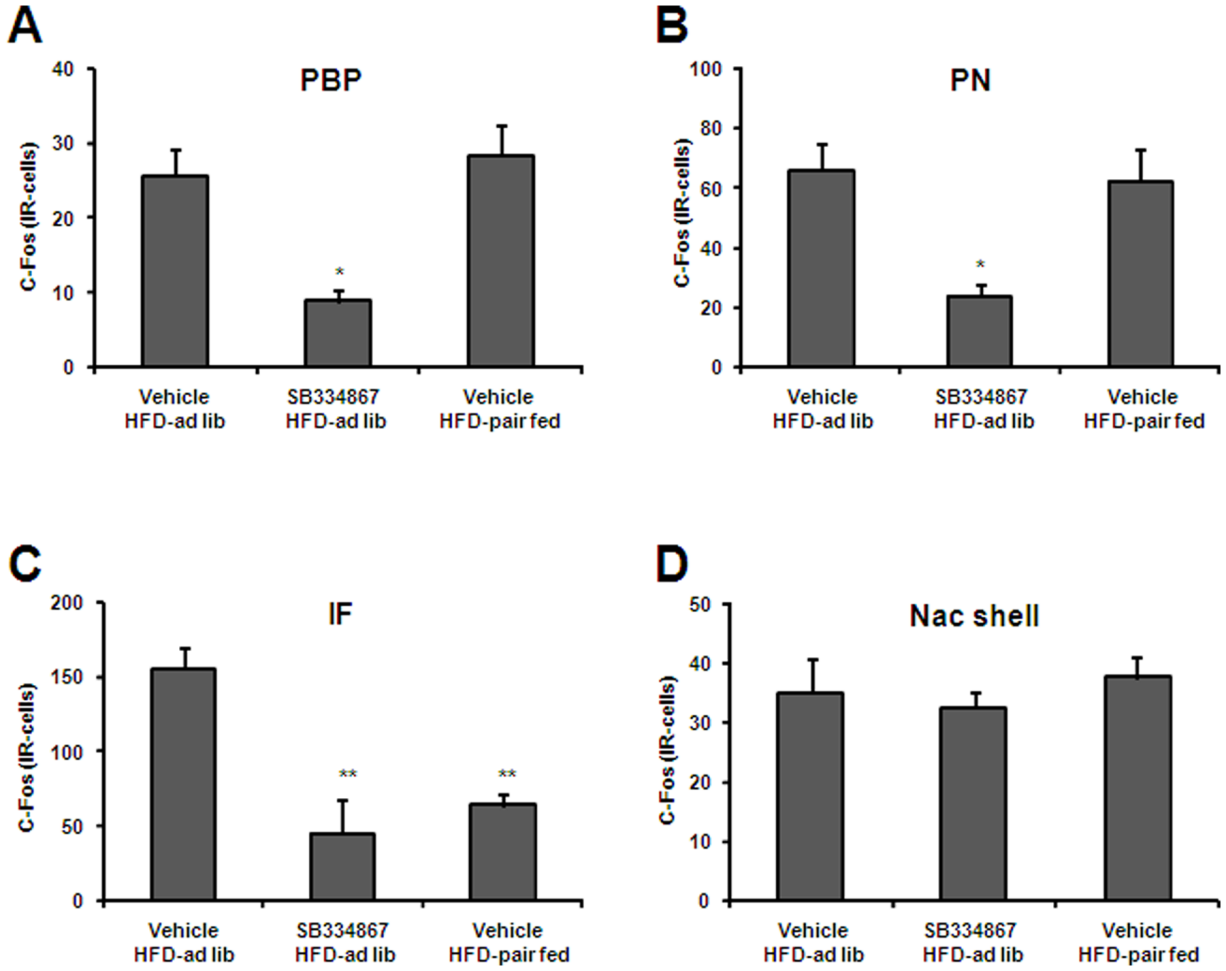
Orexin signaling blockade reduces HFD-induced increase of c-Fos in the VTA and not in the NAc. Panels **A-D** show quantitative analysis of c-Fos staining in specific PBP (A), PN (B) and IF (C) sub-regions of the VTA, and in the NAc shell (D) of vehicle-HFD-*ad lib*, SB334867-HFD-*ad lib* and vehicle-HFD-pair-fed groups. Histograms depict the total number of c-Fos-IR neurons, expressed as cells per coronal section. Values are the mean±SEM. *, p<0.05 vs. vehicle HDF-*ad lib* group. **, p<0.01 vs. vehicle HDF-*ad lib* group.

To determine if orexin signaling was required for acute HFD consumption, the orexin receptor 1 selective antagonist SB-334867 was administrated to *ad lib* fed mice that were subsequently exposed to HFD. SB-334867 treatment reduced food intake (212±27 *vs*. 119±11 mg, p<0.01 vs. vehicle-treated group), and, as a consequence, an extra pair-fed group was added to the experiment (Figure 6). SB-334867 treatment significantly affected the number c-Fos-IR cells in all three sub-regions of the VTA [PN: F(2,21) = 5.868, P = 0.0095; PBP: F(2,21) = 6.230, P = 0.0075; IF: F(2,20) = 8.396, P = 0.0023] while it did not affect HFD-induced increase of c-Fos in the NAc shell (Figure 7A-D). In particular, 66.0±9.4, 23.8±4.0 and 62.4±10.6 c-Fos-IR cells were detected in the PN sub-division of the VTA of vehicle-treated, SB-334867-treated and vehicle-treated pair-fed groups, respectively, and 25.6±3.6, 8.8±1.6 and 28.4±4.0 c-Fos-IR cells were detected in the PBP sub-division of the VTA of vehicle-treated, SB-334867-treated and vehicle-treated pair-fed groups, respectively. Thus, SB-334867 treatment significantly reduced the number of c-Fos-IR cells in the PN and PBP sub-regions of the VTA (P<0.05 vs. vehicle-treated group). As compared to the vehicle-treated group, total number of c-Fos-IR cells in the PN and PBP sub-regions of the VTA failed to decrease in vehicle-treated HFD-pair-fed group. In the IF sub-division of the VTA, 155.2±26.4, 44.8±4.0 and 64.0±8.0 c-Fos-IR cells were detected in vehicle-treated, SB-334867-treated and vehicle-treated pair-fed groups, respectively. A significant decrease of the number of c-Fos-IR cells in the IF sub-region of the VTA was found in both SB-334867-treated and vehicle-treated pair-fed groups (P<0.01 vs. vehicle-treated group). Of note, SB-334867 treatment also failed to affect HFD-induced increase of c-Fos in the NAc core, CeA and LHA (not shown). These data indicate that the acute HFD-induced activation of PN and PBP neurons depends on orexin signaling.

### LHA orexin neurons responsive to acute HFD innervate the VTA

**Figure 8:**
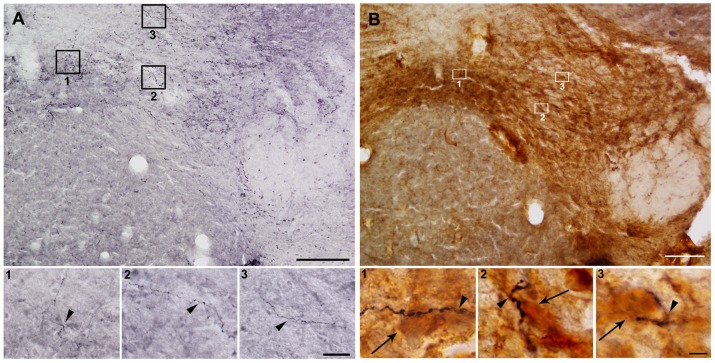
LHA orexin neurons innervate the PBP, PN and IF sub-regions of the VTA. Panel A and B show representative photomicrographs of VTA brain sections subjected to immunohistochemistry using either anti-orexin (purple/black staining) antisera alone or a combination of anti-TH (brown staining) and anti-orexin (purple/black staining) antiserum, respectively. Upper and bottom photomicrographs for each panel show low and high magnification images, respectively. Panels 1 show the IF, panels 2 show the PN, and panels 3 show the PBP sub-region of the VTA. Arrowheads point to orexin-IR fibers and arrows point to TH-IR cells contacted by orexin-IR fibers. Scale bars, 200 µm (low magnification), 20 µm (high magnification A), and 5 µm (high magnification A).

In order to identify a neuroanatomical substrate of orexin signaling within the VTA, the orexin fibers within all three sub-regions of this nucleus were quantified. Orexin-IR terminals with characteristic bouton morphology were observed in all three sub-regions of the VTA (Figure 8A). The relative density of orexin-IR fibers in the PN, PBP and IF sub-regions of the VTA was 46.3±4.2, 48.2±6.4 and 43.6±2.2 OD/100X field, respectively (p>0.05). In order to determine if orexin signaling differentially affected TH-IR neurons in the VTA sub-regions, we performed TH and orexin co-staining. Orexin-IR terminals making close contacts with TH-IR neurons were found in the three sub-regions of the VTA (Figure 8B). Quantitative analysis indicated that 84.6±8.8, 86.7±6.6 and 95.9±8.7 TH-IR neurons had apparent contacts by orexin-IR fibers in the PN, PBP and IF sub-regions of the VTA, respectively (p>0.05). Apparent appositions were also observed in TH-IR dendrites.

**Figure 9:**
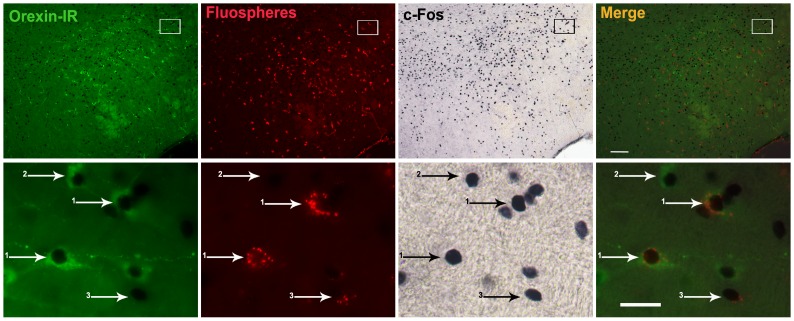
Acute HFD increases c-Fos in orexin neurons of the LHA projecting to the VTA. Upper sets of panels depict low magnification photomicrographs of the LHA of mice with stereotaxic injections of FluoSpheres in the VTA and allowed to eat HFD. Right column of images shows merge of orexin-IR (green fluorescent staining), FluoSpheres (red fluorescent), and c-Fos (black/purple staining) signals. Bottom set of panels show high magnification photomicrographs of the area marked in low magnification images. Numbered arrows point to labeled cells as follows: 1-triple-labeled cell; 2- double c-Fos/orexin-IR cell, negative for FluoSpheres; 3-double c-Fos/FluoSpheres cell, negative for orexin. Scale bars: 50 µm (Upper panels), 20 µm (Bottom panels).

Finally, we tested if orexin neurons sending their projections to the VTA were responsive to acute HFD. For this purpose, mice were subjected to red FluoSpheres injection in the VTA, exposed to HFD and then used for further double c-Fos and orexin staining. Animals used for analysis showed microinjected FluoSpheres in all three subdivisions of the VTA. Neurons doubly labeled for FluoSpheres and orexin-IR in the LHA were distributed through the whole nucleus and represented 18.4±2.1% of all orexin cells (Figure 9). Quantitative analysis indicated that 93.2±2.9% of LHA neurons doubly labeled for FluoSpheres and orexin-IR were positive for c-Fos. Also, 18.8±5.5% of single FluoSpheres-labeled neurons of the LHA were positive for c-Fos. Thus, acute HFD activates orexin neurons of the LHA that innervate the VTA.

## Conclusions

In the current study we provide evidence that acute HFD consumption recruits centers of the mesolimbic pathway including neurons of the VTA, NAc, CeA and LHA. Remarkably, we found different responsiveness of PBP, PN and IF sub-regions of the VTA to acute HFD consumption. In terms of the neuronal circuit recruited by acute HFD, we show that orexin signaling is necessary for the spontaneous consumption of HFD and for the HFD-induced activation of the mesolimbic dopamine system. In support of this possibility, we show that HFD-responsive orexin neurons of the LHA innervate the VTA.

Current findings support the notion that brain circuits driving motivation to consume a palatable food are a powerful system that can override homeostatic signals [4]. In our experimental test, mice exposed to a HFD pellet spontaneously consumed ∼1.22 kcal, which represents nearly a fifth of their daily food intake, in a short period of time. Of note, satiated mice were exposed to HFD at a time of the day when spontaneous food intake is minimal and while they remained with free access to RC. Thus, this spontaneous consumption of HFD appears to be mainly due to the palatable nature of the stimulus and involve hedonic aspects of eating. In support of this possibility, we observed a strong activation of different nuclei of the mesolimbic system. Importantly, acute HFD resulted in the same profile of c-Fos activation in the mesolimbic pathway in mice that had been previously exposed 2 h to HFD in the previous day (data not shown). Thus, neophobia (i.e. aversion to approach a novel stimulus) or novelty seeking (i.e. enhanced specific exploration of a novel stimulus) seems to play a minor role in our observations. This could be due to the minimal differences in the overall appearance of the HFD and RC pellets. Also, HFD and RC pellets were prepared with the same ingredients though in different proportion. We cannot completely rule out that some degree of neophobia exists in our experimental paradigm. However, the large amount of food intake in HFD-*ad lib* group and the lack of activation of brain nuclei known to be activated in response to a novel gustatory stimulus [34] suggest that neophobia, if present at all, is transient and minimal. Thus, we propose that the current behavioral test is a suitable model to investigate the neuronal circuits and molecular mechanisms regulating hedonic-related eating behaviors.

It is interesting to stress that we included an experimental group consisting of mice exposed to an amount of HFD weight similar to that eaten by control mice exposed to RC. This HFD-pair-fed group was used to distinguish potential effects of the HFD itself from those of higher food intake. Although RC and HFD were not isocaloric, the amount of calories consumed by HFD-pair-fed and control mice were statistically indistinguishable. Thus, HFD-pair-fed and control mice consumed similar amounts of food, expressed either as food weight or calories. HFD *ad lib* and HFD-pair-fed groups showed similar acute HFD-induced activation of most neuronal populations of the mesolimbic pathway, with the exception of the IF neurons of the VTA that were not activated in the HFD-pair-fed group (see below). Thus, it seems that the acute activation of the mesolimbic pathway is mainly mediated by the HFD itself, rather than the eaten weight or the caloric content.

We show that HFD-mediated orosensory stimulation is required for the mesolimbic pathway activation since intragastric administration of HFD failed to increase c-Fos in most of the brain areas under study. Recent studies suggest that dietary fat sensing would involve fatty acid receptors located in the tongue [35]. Then, orosensory taste information is transmitted to the NTS, which directly or indirectly recruits neuronal nuclei in the hindbrain (i.e. PBN), midbrain (i.e. VTA) and forebrain (i.e. NAc and LHA) [36], [37]. Thus, it is possible to hypothesize that the HFD-mediated orosensory stimulation due to the high levels of fatty acids present in the diet, results in the activation of neuronal circuits that end up recruiting the mesolimbic pathway. Post-oral effects of HFD have been proposed to result in overconsumption because fat is less satiating than other macronutrients [38]. In addition, it has been shown that the rewarding value of HFD is determined by its post-oral nutritive effects [39]. Current data stress the importance of the HFD-induced oral stimulation in the acute activation of the mesolimbic pathway, but it does not refute post-ingestion effects of HFD on its rewarding value. One intrinsic limitation of intragastric gavage is that it may cause unusual gastric distention and change the dynamics of nutrient entry to the intestine. In addition, stress associated to the animals handling may mask physiological responses to nutrients. However, this experimental strategy is a valuable tool to dissociate the impact of oral and post-oral signals as demonstrated by previous studies showing that intragastric infusions of glucose can stimulate dopamine system, independently of oral stimulation [40], [41].

Here we show that VTA dopamine neurons are significantly activated in HFD-exposed mice. The initial site of action for addictive drugs is known to be predominantly the VTA neurons of the mesolimbic circuit, which then influences many behaviors related to drug addiction [11]. In contrast, the role of VTA neurons in hedonic-driven food consumption is still a matter of debate [10], [13], [14]. It has been proposed that VTA dopamine neurons are responsive to pleasurable foods, or cues that predict it, and also able to promote behaviors directed towards food consumption [5], [42]. VTA contains multiple anatomically and functionally diverse sub-regions [8], [9], [31], [32], [43]. Here we have analyzed c-Fos expression within the VTA sub-regions where dopamine neurons are enriched [8] and found a differential response to the spontaneous HFD consumption. In particular, acute HFD caused a significant activation of dopamine neurons of the PN and PBP sub-divisions independently of the amount of HFD ingested. In contrast, activation of dopamine and non-dopamine neurons of the IF sub-division of the VTA occurred only in mice that over-consumed HFD and the degree of activation correlated with the amount of HFD ingested. The finding that HFD-pair-fed group showed no activation of IF neurons, regardless of their motivation for HFD eating, suggests that these neurons are responsive to the amount of HFD ingested rather than triggering its consumption. Importantly, sub-divisions of the VTA also show differences in terms of their projections and neuronal targets [8]: the PBP and PN dopamine neurons project to the ventrolateral striatum, including the lateral part of the NAc shell and NAc core, while the IF neurons selectively project to the medial part of the NAc shell. Thus, it is possible to hypothesize that sub-divisions of the VTA participate in diverse aspects of hedonic eating. Of note, sub-divisions of the VTA have been shown to respond differently to drugs of abuse, including opiates and alcohol [44]–[47].

Acute HFD leads to a strong activation of LHA orexin neurons that is required for the activation of VTA neurons and the full induction of food intake. The LHA is a key brain area that integrates gustatory and visceral information and then modulates different functions, including feeding [17]–[19]. LHA Orexin neurons are involved not only in the regulation of homeostatic food intake itself but also in food reward-related behaviors, including hedonic and motivational components of eating [17]–[19], [25]. In this regard, LHA orexin neurons project to dopamine neurons of the VTA, where orexin receptors are highly expressed and orexin activates both dopamine and non-dopamine neurons [17]–[22]. Orexin signaling is relevant for hedonic eating, as suggested by the finding that orexin-1 receptor antagonism attenuates HFD consumption in rats subject to forced caloric satiation [28]. Interestingly, the hypophagic effect of orexin-1 receptor antagonism occurs exclusively on high fat diet intake and does not affect RC intake [25]–[28]. It has been hypothesized that orexin signaling selectively enhances potentiation of glutamatergic synaptic transmission in the VTA for highly salient appetitive reinforcers [27]. However, the reason of the specificity of orexin signaling regulating HFD intake is currently unclear. Here we show that spontaneous consumption of HFD was partially blocked in mice administered an orexin 1 receptor antagonist. Thus, we have not only confirmed previous studies but also shown that even spontaneous consumption of HFD in *ad lib* fed mice requires orexin signaling.

Here, we also show that orexin signaling blockade reduced c-Fos induction in the VTA suggesting that orexin neurons are involved in the activation of the mesolimbic pathway. Indeed, we show that most LHA orexin neurons projecting to the VTA increased c-Fos in response to acute HFD. Thus, we conclude that acute HFD may indirectly engage the dopamine neurons of the mesolimbic system by initially targeting orexin LHA neurons which, in turn, project to the VTA. The estimations of orexin-IR fibers density and number of TH-IR neurons contacted by orexin-IR fibers within each VTA sub-region showed no differences. Thus, the mechanisms mediating the differential activation of dopamine neurons of the IF sub-region of the VTA in response to acute HFD will require further studies. Interestingly, we also found VTA innervating neurons of the LHA responsive to acute HFD that were negative for orexin-IR. These LHA non-orexin neurons responsive to HFD may include melanin-concentrating hormone-producing or also neurotensin-producing neurons, which have been shown to reinforce the consumption of calorically dense foods [48], [49]. Of note, a potential involvement of orexin signaling in the VTA mediating the acute stimulation of HFD consumption does not rule out that other neuronal targets of the orexin neurons also participate in this mechanism [22]. Indeed, orexin neurons project to other centers of the mesolimbic pathway known to mediate motivated behaviors, such as the prefrontal cortex, where orexin receptors are highly expressed [50]–[52]. Future studies, using selectively blockade of orexin 1 receptors in these orexin targets, are required in order to elucidate the circuit activated by acute HFD consumption.

Acute HFD leads to an activation of NAc, independent of the orexin signaling. The NAc is strategically positioned to translate the affective assessment of food into feeding behaviors since it receives critical orosensory information and then projects to hypothalamic and midbrain areas that contribute to the motor expression of feeding [12], [16], [53]. The NAc contains two functionally different sub-regions: NAc core and NAc shell [18]. The NAc core projects to the basal ganglia pathways to influence voluntary motor functions while the NAc shell projects mainly to subcortical limbic regions, such as the LHA and VTA, and modulates hedonic behaviors [18]. The role of the NAc in feeding behaviors is complex [16], [50], [53]. It has been shown that stimulation of NAc shell neurons by µ-opiod receptor agonists can stimulate HFD consumption by recruiting LHA orexin neurons that, in turn, would activate VTA neurons [16], [50], [53]. Here we show that acute HFD activated c-Fos expression in the NAc shell and that this effect was not blocked by an orexin receptor 1 antagonist. Thus, it is possible to hypothesize that NAc shell is one of the initial neuronal targets recruited by HFD consumption that then further engages other brain systems, including the LHA and VTA neurons. In support of this possibility, it has been shown that the hyperphagia observed in response to intra-NAc shell administration of the µ-opiod receptor agonist DAMGO is blocked by temporary inactivation of the LHA and VTA [53]. Although not a central focus of the current study, we observed that acute HFD also activates c-Fos expression in the NAc core and CeA in an orexin-independent manner.

Here, we have used a simple experimental paradigm in mice to provide evidence that clarify our understanding of the brain neuronal circuits recruited by the acute and spontaneous HFD consumption. Hopefully, this information will be helpful to the future development of new strategies for the treatment of conditions in which overconsumption of highly palatable food is observed. For instance, this current protocol could be used to study the neurobiology of binge-eating episodes, which refer to specific events of uncontrollable overconsumption in which the motivation to eat is usually aimed to obtain palatable energy-dense foods [54]–[56]. Episodes of binge-eating are characteristic of patients suffering from several eating disorders including anorexia nervosa, bulimia nervosa and binge eating disorder [57]–[60]. In addition, binge-eating episodes are observed in obese patients and even in healthy people under specific circumstances, such as stress [61]. Despite its clinical relevance, finding appropriate animal models for studying binge eating episodes has been challenging due to the fact that its etiology in humans is currently unclear [62]. In order to trigger acute food overconsumption, many animal models of binge eating involve a previous period of imposed food restriction, which also affects brain circuits regulating homeostatic aspects of food intake [55], [63], [64]. However, some evidences suggest that binge-eating episodes mainly involve hedonic-related feeding circuits [65]. Here, we show that satiated mice that spontaneously consumed a large amount of HFD in a short period of time have a strong activation of the VTA-NAc-LHA pathway. Therefore, this experimental protocol may be helpful to study the eating episodes in early stages, when the mesolimbic pathway would presumably play a key role [66].
