# Correction: Acute High Fat Diet Consumption Activates the Mesolimbic Circuit and Requires Orexin Signaling in a Mouse Model

**DOI:** 10.1371/journal.pone.0092932

**Published:** 2014-03-14

**Authors:** The PLOS ONE Staff

The image quality for panel A in Figure 2 and Figure 3 is poor in the published article.

For a better quality version of Figure 2A, see here:

**Figure 2:**
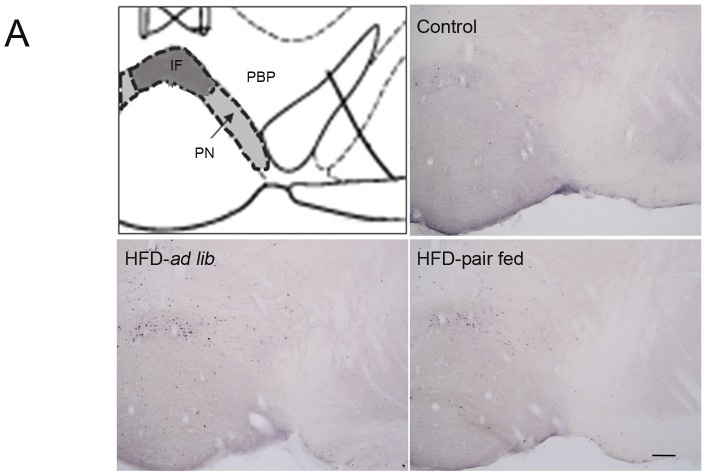
Acute HFD activates c-Fos in specific sub-regions of the VTA. Panel **A** shows a schematic diagram of VTA sub-regions in a coronal section of the mouse brain (upper left) and representative microphotographs of c-Fos (black/purple signal) immuno-staining in the VTA of control (upper right), HFD-ad lib (bottom left) and HFD-pair-fed (bottom right) groups. Scale bar: 100 μm.

For a better quality version of Figure 3A, see here:

**Figure 3:**
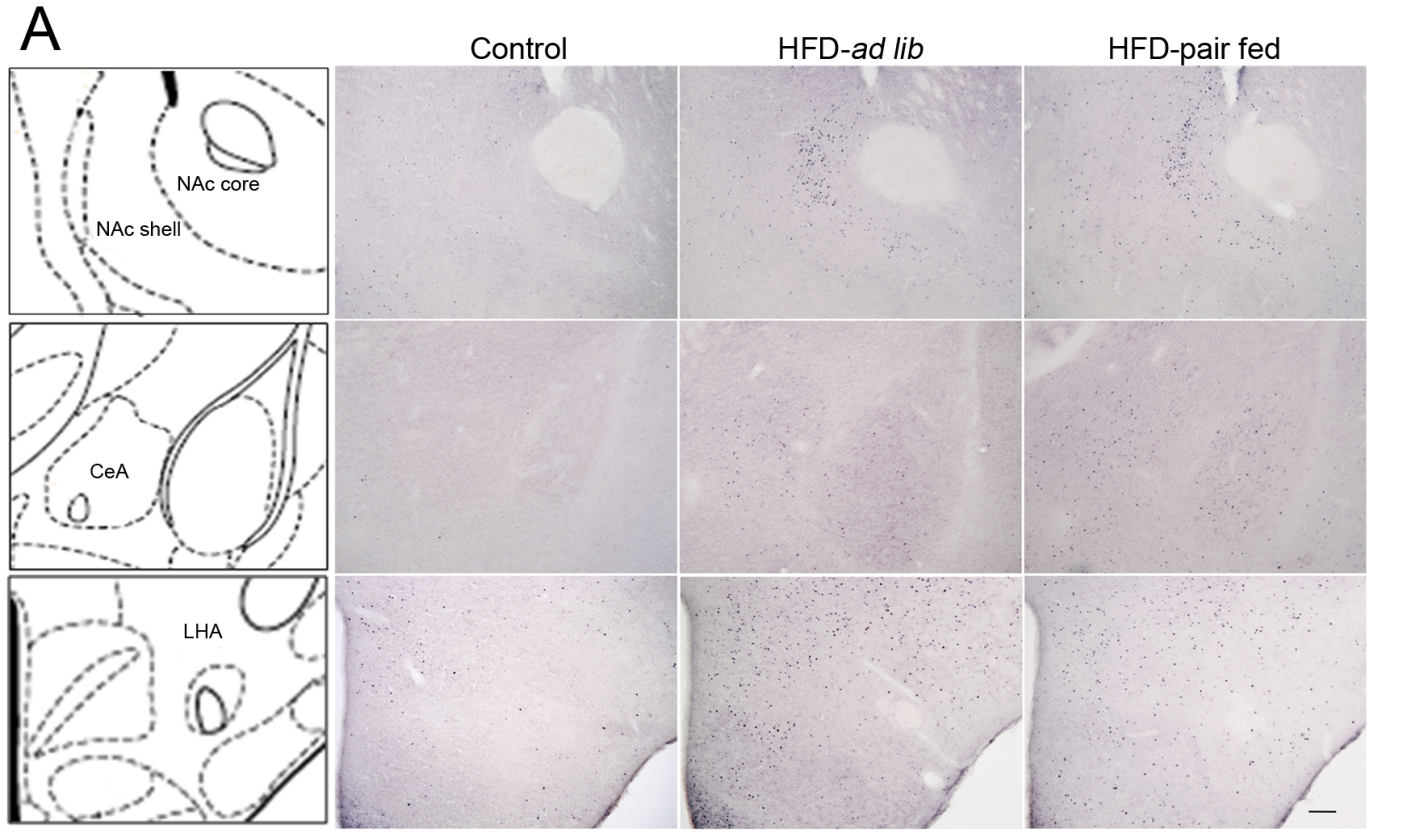
Acute HFD activates c-Fos in specific nucleus of mesolimbic pathway. Panel **A** shows a schematic diagram of the brain regions under study in a coronal section of the mouse brain (left column) and representative microphotographs of c-Fos immuno-staining of control, HFD-ad lib and HFD-pair-fed groups. Upper, middle and bottom line of images show the NAc (core and shell), the CeA and the LHA, respectively. Scale bar: 100 μm.
